# A Coalition for Change? Role Orientations in the 12th Parliament of Botswana

**DOI:** 10.1080/03057070.2024.2476901

**Published:** 2025-04-04

**Authors:** Anja Osei, Batlang Seabo

**Affiliations:** Freie Universität Berlin; University of Botswana

**Keywords:** Botswana, parliament, parliamentary reforms, role orientations, coalition for change

## Abstract

Botswana’s parliamentary democracy features a weak parliament that is ineffective in law making and executive oversight. Conventional explanations emphasise a dominant party system that emerged following independence, lack of operational independence from the executive, and the poor capacity of parliament as factors that undermine its effectiveness. Using a novel dataset that is based on interviews with Members of Parliament (MPs) on a wide range of issues, including their role orientations, this article tests several interrelated hypotheses to investigate whether there is an emerging coalition for change. The article finds that there is a group of opposition MPs that constitutes a coalition for change because they are reform oriented, discuss parliamentary affairs and exercise executive oversight. We argue that this coalition for change is marked by a connected communication structure. The study furthers our understanding of the functioning of parliament as a core institution of Botswana’s democracy.

## Introduction

Successful democracies depend on effective political institutions. By the same logic, ineffective institutions can undermine democracy. Since parliaments are crucial in providing a link between citizens and politics, their performance has direct consequences for democratic quality and popular satisfaction. Steven Fish finds a strong correlation between parliamentary powers and democratic quality: stronger legislatures lead to stronger democracies.^1^ While Fish concentrates on formal powers,^2^ Joel Barkan argues in a highly influential edited volume that the democracy-promoting functions of parliaments are connected to agency.^3^ In particular, he holds that change is promoted by coalitions of reform-oriented Members of Parliament (MPs). These ‘coalitions for change’ are composed of MPs who are interested in strengthening parliament and who actively contribute to political reform.^4^ Other MPs, by contrast, are more engaged in patronage relations and constituency service.^5^

Although Barkan’s work^6^ has been highly influential in African studies, his theory of role orientations of African MPs has not been tested in later empirical studies. This article fills this gap: using data collected in the 12th Parliament of Botswana in 2022 and 2023, we demonstrate that MPs differ in their behaviour and preferences. These differences can be grouped. There are MPs who are significantly more interested in parliamentary affairs than others. The same MPs tend to place a comparatively high value on executive oversight and criticise institutional inefficiency. Moreover, they tend to place less emphasis on constituency service than others and have greater political experience in parliament. Following Barkan, we characterise this group as a coalition for change, but we add an important level of analysis, namely that of coalition-building as a communicative structure. While the chapters in Barkan’s edited volume implicitly assumed that reform-oriented MPs communicated with each other, they did not show this empirically. Based on a dataset containing information on political discussion networks we are able to capture this aspect.

We seek to answer two questions:

- Do we find an integrated communication structure that links MPs who are interested in parliamentary reform?
- By which attributes are these MPs characterised? What do they have in common?

Hence, the article makes a theoretical and an empirical contribution: it expands this theory by including social network relations between MPs and provides an empirical test in the case of Botswana.

The article is organised as follows: the next section reviews the literature on conventional explanations regarding African parliaments. Thereafter, the article presents a theoretical discussion of Barkan’s coalition for change, followed by a section on the parliament of Botswana focusing on its institutional weaknesses and reforms. Several interrelated hypotheses are then stated in the next section, drawing on Barkan’s theory. A discussion of the data and methodology follows, leading up to the analytical part of the article. We then draw conclusions and implications of the study for future research.

## African Parliaments: The Limits of Conventional Accounts

For some time, African parliaments were among the least researched institutions on the continent. Recently, however, legislatures have received growing scholarly interest.^7^ Consistent with the conventional wisdom that African countries have no or weak institutions,^8^ much of the literature on African parliaments has focused on institutional weaknesses and clientelist practices.^9^ Since voters are believed to prefer candidates who can credibly claim to bring developments to their home communities, MPs tend to place a priority on constituency service rather than other functions like law making or executive oversight.^10^ Thus, the policy-making function is weakly developed. In the absence of programmatic competition, policy congruence and effective feedback mechanisms, MPs outbid each other in campaign promises for public and private goods.^11^

There is, however, considerable variation across and within countries. Robert Mattes and Shaheen Mozaffar find that the electoral system impacts MPs’ behaviour: MPs elected in single-member districts are more likely to focus on constituency service than MPs in countries that use party list proportional representation systems.^12^ In Ghana, for example, two strong parties structure patterns of clientelism.^13^ Martin Acheampong also finds that the time individual MPs spend in their constituencies varies widely.^14^ Moreover, there is variation in who is targeted: MPs in uncompetitive constituencies tend to channel favours only to party delegates, whereas MPs in contested constituencies build relations to the wider population.^15^ For Nigeria, Leila Demarest holds that MPs are relatively wealthy, but direct their resources to a very narrow group of party elites rather than constituents.^16^ Andrew Harris and Daniel Posner argue that local conditions such as the spatial segregation between supporters and opponents shapes the targeted allocation of resources from constituency development funds in Kenya.^17^ They also find that women are less clientelistic than men, and that experienced MPs – those who have served longer terms in parliament – are less likely to channel projects to their supporters.^18^ The authors explain the latter finding with the ‘offsetting effects of greater experience and the lesser need to reward one’s supporters due to distributions made during prior terms’.^19^

The variations in clientelistic behaviour interact with variations in other roles that MPs fulfil. Demarest shows that Nigerian MPs who have access to lucrative committees are also more active in formal parliamentary business, such as sponsoring bills.^20^ Similarly, Michaela Collord argues that where individual legislators in Tanzania act as powerful local patrons, they gain more autonomy from the executive.^21^ Ken Opalo finds that legislatures are strengthened when they become the arena for intra-elite bargaining.^22^

In one of the few studies on non-clientelist aspects of MPs’ behaviour, Jesper Katomero *et al*. find four groups of MPs that differ in their perception of accountability.^23^ While a number of MPs are deeply committed to holding the executive accountable, they also face practical constraints. Others are more pragmatic, or oriented towards their party or their electorate. Experimental evidence shows that information on MPs’ policy performance can contribute to better informed vote choices, but the effects depend on the salience of the policy issue and on the spread of information.^24^

All of this recent literature expands our understanding of MPs’ individual patterns of behaviour. Obviously, MPs face varying incentives to which they respond. Taking this as a starting point, we argue that MPs hold different role orientations, and that reform-oriented MPs are more likely to form a coalition for change.

## Coalitions for Change and Role Orientations

Role orientations are understood as a set of norms of behaviour.^25^ Different roles can be in conflict with each other, for example if parties and constituents expect different types of behaviour from an MP. The behaviour of individuals is thus shaped by different external stimuli, as well as an MP’s own understanding and definition of a situation, and personal characteristics such as socialisation, personality, and character.^26^ Or, as Kaare Strøm argues, role orientations are driven by preferences, but constrained by institutions.^27^ Later work has identified different roles and their effects, mostly concentrating on established democracies. Work on role orientations in new democracies or hybrid regimes and the ways in which they influence coalitions for change is relatively scarce.^28^

The most important work on role orientations as drivers of coalitions for change in Africa is Barkan’s edited volume, *Legislative Power in Emerging African Democracies*.^29^ Barkan’s starting point in the introduction to the edited volume is a trade-off between different roles that MPs can play.^30^ He defines parliamentary functions as representation, legislation, oversight and constituency service. These functions exist in tension to each other, and individual MPs prioritise some functions over others. In Africa, MPs tend to spend more time and resources in their constituencies, which comes at the expense of other functions like law making and oversight.^31^ Reformers, then, are MPs who are committed to democratisation and legislative development.^32^ They are often highly educated and young, and consider themselves a ‘new generation’ of politicians.^33^ Barkan emphasises that this group does not need to be large to make an impact, because reformers are often joined by opportunists who support certain aspects of a reform-oriented agenda.^34^ On the other hand, reformers often struggle to be re-elected because they place less effort on constituency service.^35^

From their case studies, Barkan and his colleagues develop their main argument that ‘legislative development occurs when a viable group of legislators that we call “a coalition for change” emerges to break out of these constraints to reshape the structure of incentives and the formal rules’.^36^ Although there is variation across countries in the extent to which coalitions for change exist and accomplish their goals, the findings demonstrate that the constraints facing parliament are physical, for example the lack of adequate buildings, lack of staff and financial resources at parliaments’ disposal.^37^ While the gains in legislative strengthening have remained modest in Benin,^38^ institutional capacity in Nigeria expanded thanks to some veteran members and their allies.^39^ Rent-seeking remains rampant, however.^40^ With regard to Ghana, Staffan Lindberg and Yongmei Zhou^41^ point to the weak separation of powers and the institutional incentives that are mostly reform-unfriendly. A coalition for change did not emerge in these cases, but Kenya is cited as a successful case of legislative development, initiated by a reform coalition of about 10 per cent of the MPs.^42^

Later studies have extended Barkan’s argument or even challenged it. Collord argues that elite power struggles in ruling parties can open up avenues for legislative strengthening.^43^ Opalo explains the variation between Kenya and Zambia, calling to attention the different historical relations between legislatures, the executive and the ruling party in those countries.^44^ Amy Poteete points out that the emergence of a coalition for change is only ‘half of the story’ if the executive resists legislative strengthening.^45^ Legislative empowerment can be brought about in different ways, but at the same time, successful change by a coalition of actors is not a given.

## Defining and Measuring Coalitions for Change

Leaving aside the question of what coalitions for change contribute to legislative development, there is some methodological criticism that has not yet been addressed. Barkan’s definition of coalitions for change is rather loose and stems from qualitative interviews. The interviewed MPs represent only a small fraction of the total number, and although they were balanced according to descriptive attributes like gender, ethnic origin and party affiliation, a selection bias cannot be excluded. Interviewing only a share of MPs risks oversampling those MPs that are most accessible and willing to give an interview, thus overestimating the presence of reformers. MPs who are more devoted to constituency service are often less interested in being interviewed, or have a lower chance of being selected because they spend more time in their electoral areas. This makes it difficult to generalise from the findings.

Taking the idea of the coalition seriously, it is not enough that there is a group of like-minded MPs – these groups must also be able to communicate. While this sounds trivial, it might mark an important step from a loose group to a coalition. Following Heinrich Best and Lars Vogel, and Anja Osei,^46^ the parliament is a social space of daily interactions between legislators. They come together to exchange views, discuss politics, and perhaps form new initiatives of political action. Although Barkan does not develop a theory of legislative communication, it can be assumed that the formation of a coalition is a process that is constituted by patterns of interaction between different MPs. Only in their conversations and perhaps speeches on the floor can they recognise peers that support reform. Therefore, coalitions for change emerge through political exchange. Without a network of interaction, like-minded MPs will still be isolated.

Instead of explaining outcomes, therefore, this article will theoretically and empirically advance the concept of coalitions for change and explore it using data from Botswana. Theoretically, we advance the concept of a ‘coalition for change’ from a loosely defined group of like-minded people to a stricter understanding of a coalition as a *connected communication structure* within parliament. Since we are providing the first empirical test outside Barkan’s own work, we opt for a data-driven, yet exploratory approach. In a first step, we will identify MPs in our data who discuss parliamentary reform, and analyse the degree to which those MPs form a connected communication structure. In a second step, we will explore the attributes of those MPs and link them to Barkan’s theory. Our approach will advance the concept theoretically and empirically and provide a framework that can be further tested in future studies.

## Parliament and Democracy in Botswana

Botswana’s parliamentary democracy is regarded as a torchbearer in sub-Saharan Africa.^47^ The country is one of the few to maintain a multiparty system in a region that was characterised by authoritarian regimes and one-party systems following independence from former colonial powers.^48^

Democratic practice, prudent economic management, fairly effective democratic institutions, and selection of the governing elite through elections are cited as the hallmarks of Botswana’s democracy.^49^ However, although Botswana is widely regarded as a beacon of democracy on the continent and has a two-term limit for the presidency, until 2024, there was only one party in government since the country’s first elections in 1965, the Botswana Democratic Party (BDP). Nevertheless, the consistent and uninterrupted record of periodically holding elections, with low levels of electoral fraud and violence, has led scholars to conclude that the electoral process has been successfully implemented and has helped to reinforce and maintain the separation of powers between the legislature, the executive and the judiciary.^50^ Moreover, Botswana was able to achieve this political feat without violence as all political parties accepted the legitimacy of the BDP to govern following elections.

The Constitution provides for the existence of the three fundamental state institutions commonly found in modern democratic societies.^51^ By African standards, these institutions are relatively functional and together with a stable political climate have contributed to Botswana’s rapid economic development. In line with this argument, Balefi Tsie argues that ‘state institutions matter in development outcomes and also in nourishing and supporting democratic practices’.^52^

Like parliaments elsewhere in the world, Botswana’s parliament performs several functions. These functions have been extensively described by scholars, thus we briefly review them to provide context. First, Section 86 of the Constitution empowers parliament with ‘making laws for the peace, order and good government of Botswana’.^53^

The other function of parliament is to approve the budget presented by the Minister of Finance.^54^ Parliament’s control over public finances includes approval of supplementary budget requests by ministries. Moreover, Section 50 of the Constitution tasks parliament with the responsibility of oversight over the executive. Among others, oversight is exercised through parliamentary question time, select committees and commissions of inquiry.^55^ The election of the president and appointment of cabinet ministers are also part of the duties of parliament, and parliament is empowered to pass a vote of no confidence in the president and his government (see Section 92 of the Constitution of Botswana).

However, Botswana’s parliament exhibits several weaknesses and constraints seen in other parliaments in the sub-Saharan African region.^56^ These constraints have implications for its proper functioning, leading analysts of Botswana’s political system to call for reforms that would strengthen parliament.

We turn to review briefly major weaknesses of parliament, reform motivations, and reform attempts made to enhance its functioning.

## The Need for Reform and Reform Attempts in Botswana

Before we test for a coalition for change, this section discusses some of the previous reform attempts to strengthen Botswana’s parliament. We review the literature on the weaknesses of parliament and proposed reforms as a prelude to testing our expectation of the existence of a coalition for change. As Barkan reminds us, a coalition for change is a group of reform-oriented MPs.^57^ To begin with, several studies conclude that Botswana’s parliament is beset by the country’s political system, just like elsewhere in Africa where Barkan and his colleagues observed that political systems that emerged following independence undermine the oversight role of parliaments.^58^

Scholars have documented the political ramifications of Botswana’s long-standing, dominant party system.^59^ The many years of one-party dominance under the BDP rendered ineffective the oversight role of parliament and this was also reflected in the composition of parliament.^60^ For Kenneth Good, it was this predominance that reinforced elitism, centralised political power and shielded the executive from accountability.^61^ For instance, Section 47 of the Constitution of Botswana empowers the president to appoint ministers and assistant ministers, among other key appointments. While there are several advantages of executive-parliament fusion, this dispensation undermines the effective oversight of parliament due to the inextricable relationship between the executive and parliament in two significant and related ways. First, the principle of collective responsibility binds all cabinet ministers and curtails their ability to exercise oversight upon their fellow cabinet ministers, leading some scholars to conclude that ministers are accountable to themselves.^62^ Second, the dominance of BDP legislators in parliament means that more than half of them serve as cabinet and assistant cabinet ministers, leaving only a small proportion of backbenchers able to exercise any meaningful oversight along with other opposition MPs.

Moreover, as mentioned earlier, like other parliaments in Africa, the parliament in Botswana is regarded as a rubber-stamping institution: lacking effectiveness in passing legislation, rather it just approves it.^63^ The limitation is that parliament is not empowered to conduct research, and MPs generally lack legal expertise to draft bills or comprehend the intricacies of law making.^64^ Furthermore, Botswana’s executive is only indirectly accountable to parliament through civil servants.^65^ For instance, Mpho Molomo observes that a major deficit in the case of Botswana is that the president does not answer questions in the house.^66^ This is despite the fact that the president of Botswana is elected by parliament rather than the people.^67^ Moreover, effective oversight requires specialised committees supported by sufficient personnel.^68^ However, the dominance and overlapping membership of BDP legislators in parliamentary committees renders them weak and ineffective.^69^ David Sebudubudu and Bertha Osei-Hwedie criticise the committee system for its reliance on the assistance of administrative officials from the Office of President which compromises its proper functioning in a democratic setting and undermines the independence of parliament.^70^ Related to this, the limited independence of parliament from the executive helps to explain parliament’s lack of an independent budget.^71^

It is on this basis that proposals for reforms to make parliament more independent and effective in the discharge of its functions were seen to be necessary.^72^As early as 1988, a motion was tabled in parliament to make it more independent from the executive. Recognising the weaknesses of parliament, the motion called for parliament to be detached from the executive and become an independent institution.^73^ Fifteen years later, in 2003, a study was commissioned to investigate parliamentary independence in order to address the shortfalls of Botswana’s parliament.^74^ The study recommended the following reforms:

- removal of parliament from the Office of President;
- provision of independent parliament budget;
- establishing parliamentary service commission to hire parliament staff;
- legislative reform (the amendment of Section 20 of the Constitution of Botswana so that the Clerk of the National Assembly and other offices fall under the Parliamentary Service);
- business forecast (more time to be allocated to parliament’s own business);
- developing a parliament charter and strengthening public accountability to enhance parliament’s role.

However, none of these proposed reforms were adopted. As Emmanuel Botlhale and Kebapetse Lotshwao write, the task force submitted its report to the Speaker of the National Assembly in February 2011 and only debated it in parliament in August 2011, ‘*without much achieved*’ (emphasis added).^75^

Moreover, legislators across the political divide have advocated for parliamentary reform. Notable MPs include former Speaker of the National Assembly Dr Margaret Nasha, who strove to achieve the independence of parliament from the executive, notwithstanding her then ruling-party affiliation.^76^ One of the proposed reforms to strengthen parliament is to provide MPs with legal expertise or offices to facilitate the law-making function of parliament.^77^ Moreover, David Sebudubudu and Mokganedi Botlhomilwe^78^ write that Botswana’s parliamentarians admitted that parliament lacks independence and needs to be reformed and empowered to enable it to effectively discharge its constitutional mandate. This seems to be in line with Barkan’s coalition for change thesis. If MPs with a preference for institutional strengthening come together, they form what Barkan terms a coalition for change. While these coalitions are largely made up of ‘opportunists’ interested mainly in increasing their own salaries, perks and careers, they are driven by ‘reformers’ who are ‘intent on transforming their institution from a weak rubber stamp of the executive into a modern autonomous legislature’.^79^ More recently, MPs across the political divide have supported a motion to increase their salaries and allowances.^80^ However, it is pertinent to note that, in line with Barkan, some opposition MPs acted as reformers by criticising some aspects of the Salaries and Allowances Bill.^81^ Notably, the opposition has amplified the need for reforms to strengthen parliament. For instance, the Leader of the Opposition in the 11th Parliament reiterated that parliament’s powers and independence should be enhanced.^82^ This provides us with a basis to test Barkan’s coalition for change thesis in the context of Botswana’s parliament using novel data collected from MPs. In the section that follows, we outline several hypotheses we set out to test and then discuss the research design and methods we use to test these hypotheses.

## Data Collection and Empirical Approach

We use the case of Botswana as a most likely case.^83^ It is an important requirement for our test that Botswana is one of the most democratic countries in Africa, because it enables political actors to pursue their reform goals relatively unrestricted. In authoritarian countries, by contrast, reform activities can be limited by repression and co-optation. The BDP was still the dominant party at the time of our data collection, but the opposition had actively pushed for reform, as detailed above. Locating a coalition for change in Botswana’s 11th Parliament offers us another advantage given the split of the BDP and the formation of Botswana Patriotic Front (BPF), sponsored by former president Ian Khama. These political reconfigurations in Botswana’s party system raise implications for possible existence of a coalition for change. Under such circumstances, it is very likely that reform-oriented MPs in parliament come together to discuss their goals. If no coalition for change is found in a most likely case, however, the theory will be weakened or will need to be modified. Our article explores data collected in the Parliament of Botswana in 2022 to answer our research questions. The first question is concerned with the social network aspects of the coalition for change: do we find an integrated communication structure that links MPs who are interested in parliamentary reform? The second question looks at the attributes by which MPs in this coalition are characterised.

The case of Botswana is part of a larger project on parliaments and democracy in Africa that sought to understand communication patterns and structures of political discussion in parliaments. The questionnaire is the same for all cases and includes different types of data: biographical data, relational data on MPs’ interactions, and data on values and attitudes. From these data, we not only know which MPs talk to each other, but also which topics are discussed. In addition, biographical information from the survey is used to shed light on the social and demographic composition of the legislature. Finally, the study also contained questions on MPs’ priorities, values and attitudes, which gives us important information about their role orientations. Data collection in Botswana began in August 2022.^84^ In total, 52 of the 63 MPs in the 11th Parliament of Botswana participated in our survey, giving us a response rate of 82.5 per cent.

In the empirical analysis, we follow a two-step approach: we first look at the structure of communication. Our working definition of a coalition for change includes MPs who discuss parliamentary issues with fellow MPs. In the second step, we look closer at the socio-demographic attributes and values of these MPs. The method is exploratory in nature, combining a number of tools to uncover differences in the behaviour of MPs. Our approach has the advantage of directly modelling the idea of a coalition as an interaction structure and then describing the differences between members and non-members of this coalition. We therefore come very close to operationalising the content of the theoretical concept that Barkan developed. Moreover, we work with a much more representative dataset which allows us to draw more generalisable conclusions.

## A Coalition for Change in Botswana: The Structure of Communication

Relational data were collected using a name generator question: MPs were asked: ‘[l]ooking back over the past six months, with which other MPs in the Parliament of Botswana have you discussed political issues? Please give me their names’. Each MP named four to five MPs with whom he or she had discussed political issues. We then followed up with a question on the topic that was discussed in each dyad: ‘what was the topic of this discussion?’ It turned out that MPs discuss a large number of different issues, which we then coded inductively from the data into broad topic categories. The category ‘parliamentary affairs’ includes verbatim responses, such as: ‘[we discussed the] role of parliamentary committees’, ‘how to manage attendance in parliament’, ‘how to improve parliamentary effectiveness’, ‘regime change and parliamentary debate strategies’. We assume that all MPs who discuss parliamentary affairs have an interest in the role of parliament as an institution. Hence, all MPs discussing parliamentary affairs are candidates for membership in our coalition for change. In this way, we use relational data to approximate Barkan’s idea of a ‘coalition’.^85^

**Figure 1.**
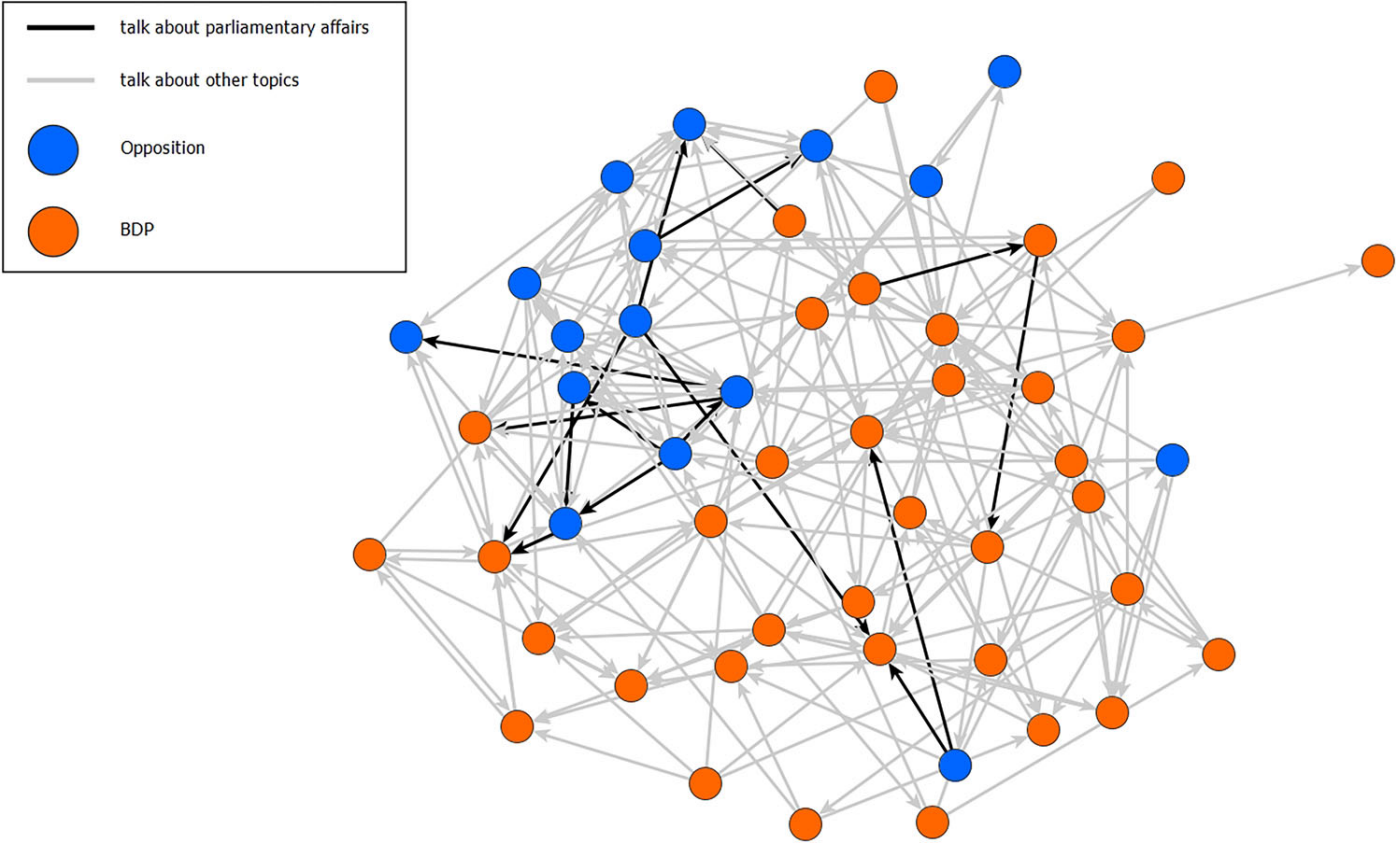
Network of political discussions in the Botswana parliament.

From these data we construct a network of political discussion in parliament that shows the general interaction structure. For visualisation, we use Visone,^86^ a freeware tool for social network analysis, and for the descriptive statistics and data management we use the Statnet R package.^87^ To test for the connected structure of our coalition for change, we plot the networks as graphs (see Figure 1).

The network of political discussions that they form is composed of 52 nodes (representing the 52 MPs) and 226 links. Eighteen MPs report taking part in discussions about parliamentary affairs. Of the 18 MPs, eight belong to the BDP and ten to the opposition. The composition of the coalition for change is significantly different from the overall composition of parliament (X-squared = 5.0472, p-value = 0.02). They form 16 topic-related links of which nine connect two opposition MPs, only two connect two BDP MPs, and five connect MPs of the ruling party and the opposition.

The network is shown in Figure 1. BDP MPs are represented by orange circles and opposition MPs by blue circles. The darker linking lines are those links that mark our coalition for change. It is immediately obvious that the coalition for change is dominated by opposition MPs. Second, the links form a connected subgroup.

**Figure 2.**
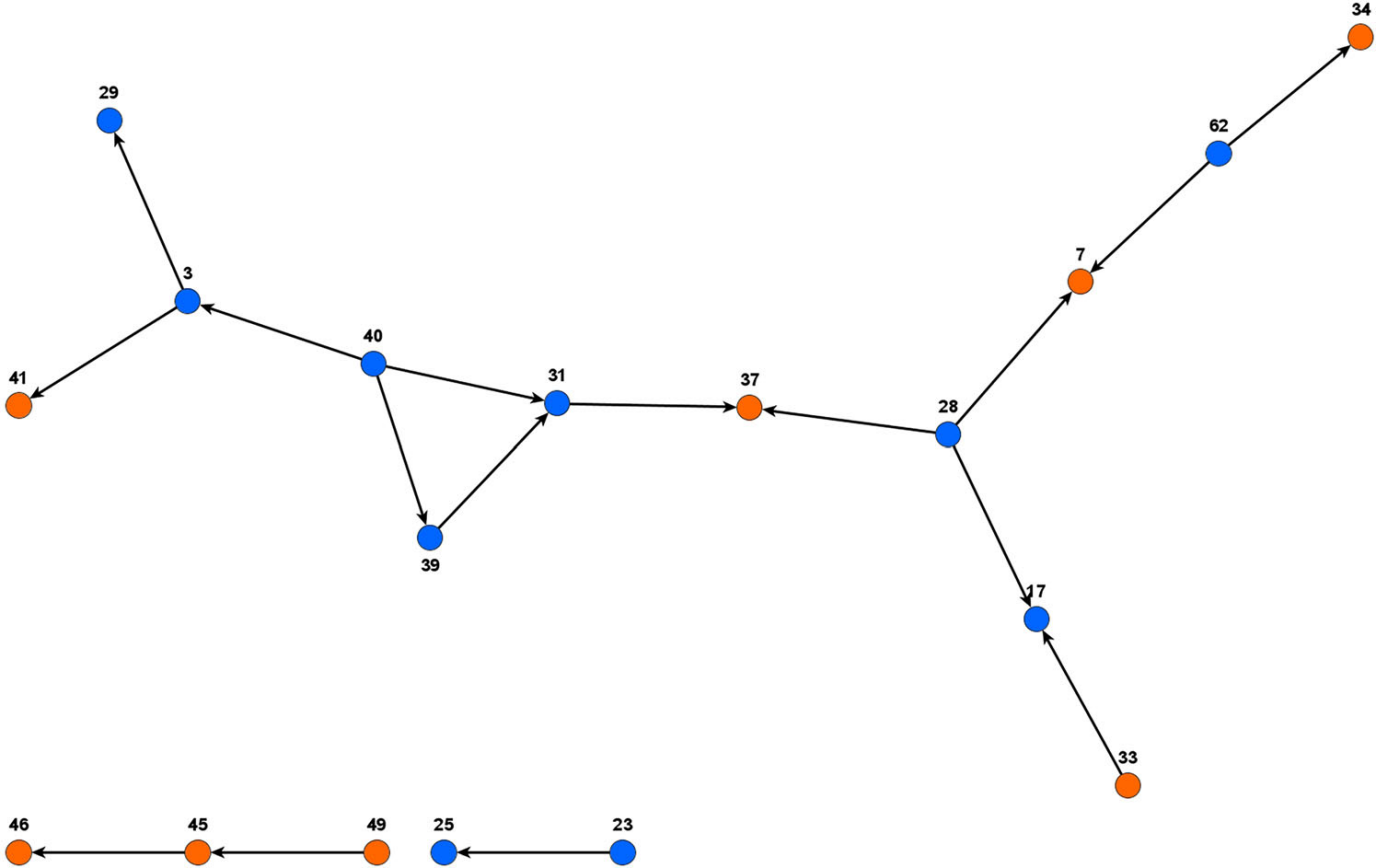
Subgroup of Botswana parliament network showing only discussion of parliamentary affairs. The numbers denote MPs by ID numbers that anonymise data collected in the research.

The latter fact becomes even more obvious when we extract a subgraph that shows only the discussion of parliamentary affairs (see Figure 2). The numbers denote MPs’ ID numbers instead of names: since we collected highly sensitive personal data, we present our analysis in an anonymised way. Figure 2 contains only those MPs who discuss parliamentary affairs (those that are connected by bold lines in Figure 1).

There is a largely connected discussion structure. In social network analysis, components are portions of a network that are disconnected from each other. Figure 2 shows three components, a big one which connects a larger number of nodes, and two small components. Opposition MPs with ID 31, 39 and 40 in the big component form a closed triangle, which is also known as a 3-clique. This is a graph structure of three people in which all possible dyads are connected. Structurally, we see that the MPs who discuss parliamentary affairs are in fact connected to each other.

The position of the BDP is interesting, however. Actors 37 and 7 are both BDP members who are nevertheless central to the discussion structure. Interestingly, both have only incoming ties: this shows that they are not initiating the discussion about parliamentary affairs themselves, but are approached by opposition MPs. One of them is the government chief whip, and one of the thematic discussions revolves around the management of attendance in parliament.

It is interesting to note, however, that three BDP MPs form a small sub network (Actors 46, 45 and 49). From the verbatim responses we know that 49 and 45 talk about ‘our role as backbenchers in parliament’. Backbenchers are MPs with no front bench or formally important role in parliament. The interest of these MPs might be more self-centred and therefore differ from that of others who are more interested in a general reform of the political system.

## Characteristics of MPs in the Coalition for Change

We further theorise that MPs in this group share biographical attributes and preferences. We expect them to evaluate the work of parliament more critically, and pay less attention to constituency service but more attention to executive oversight. Moreover, we argue that they are more interested and more active in parliamentary affairs, most notably in parliamentary debates. We also control for the number of terms and for party affiliation to see whether the coalition is composed of newcomers or more experienced MPs, and whether the affiliation with the ruling party or the opposition makes a difference. According to Barkan, reform-oriented MPs are often voted out due to their low engagement in constituency affairs. We would hypothesise that the coalition for change should be mainly composed of newcomers. On the other hand, for a coalition to form, MPs should already know each other and have formed networks through mutual contact and discussion. Although we consider this alternative, we follow Barkan’s original thinking and expect that MPs in the coalition for change are mostly newcomers and mostly opposition MPs.

We use data from our survey to further investigate these relationships, attitudes and behaviour of MPs. Full details of the variables and how we coded them can be found in the Appendix. First and most importantly, we identified 18 MPs who are part of a dyad that talks about parliamentary affairs, using the binary variable we label as *talkparl*. We also identify MPs according to the binary variable *party*, which has two categories, BDP and opposition. In a dominant party system like that of Botswana the main cleavage in the party system is between the ruling party and the opposition. We further measure and code the number of terms that an MP has served in parliament in the variable *terms* to determine if newness in office makes a difference to their likelihood of joining a coalition.

We also coded responses to questions about the effectiveness of parliament in executive oversight in the variable *oversight*. MPs asked to rank a number of tasks that MPs typically fulfil by their importance. The variable items are: bringing development to the constituency; executive oversight; law making; being close to the people; and giving feedback to constituents. This enables us to identify MPs who rank executive oversight as the most important or second most important item, and those who do not. The variable oversight has two levels: it is coded as oversight_prio if the MP has ranked executive oversight as the most important or second most important item, and as no_oversight_prio if not. Finally, we also considered a number of behavioural variables – measuring the frequency with which they visit their constituencies (*goinghome*), the number of words an MP has spoken in the house during the parliamentary year 2019 (*words*), calculated from Hansard records that are available on the website of the Parliament of Botswana.^88^

We explore the relationships between these variables using multiple correspondence analysis (MCA). We use this method because, first, many of our variables are categorical, and MCA provides a good option to map and visualise this type of data. Second, we have only 52 observations. While regression analysis is possible, the small sample size would allow only for a limited number of covariates. Moreover, our approach here is more exploratory, as we are less interested in predicting a coalition for change from our data. MCA is a useful tool to explore the association between categorical variables and thereby explore the underlying variance in a dataset. In contrast to other statistical tools, the data do not need to meet assumptions about their distribution for MCA: the only condition is that the data have the format of a rectangular table with positive values in rows and columns.^89^ The analysis is performed using a number of packages for the statistical computing environment R: the packages FactoMineR^90^ and the Factoshiny^91^ app are used for the analysis. Moreover, Factoextra offers some enhanced visualisation options.

The output of MCA produces clouds of points in a Euclidean space whose distances can be interpreted. The distances between row points are the approximate chi-square distances between the row profiles, and distances between column points are the approximate chi-square distances between the column profiles.^92^ The centre, or origin, of a graph coincides with the average row and column values.^93^ Points that are close to the centre therefore take on values that are not very different from the mean row or column value. We obtain a multidimensional space, in which each category or individual can be represented as a point. The data points are then projected onto only two dimensions, retaining the most important information while reducing the complexity of the data (for more on biplot interpretation, see Greenacre^94^).

**Figure 3.**
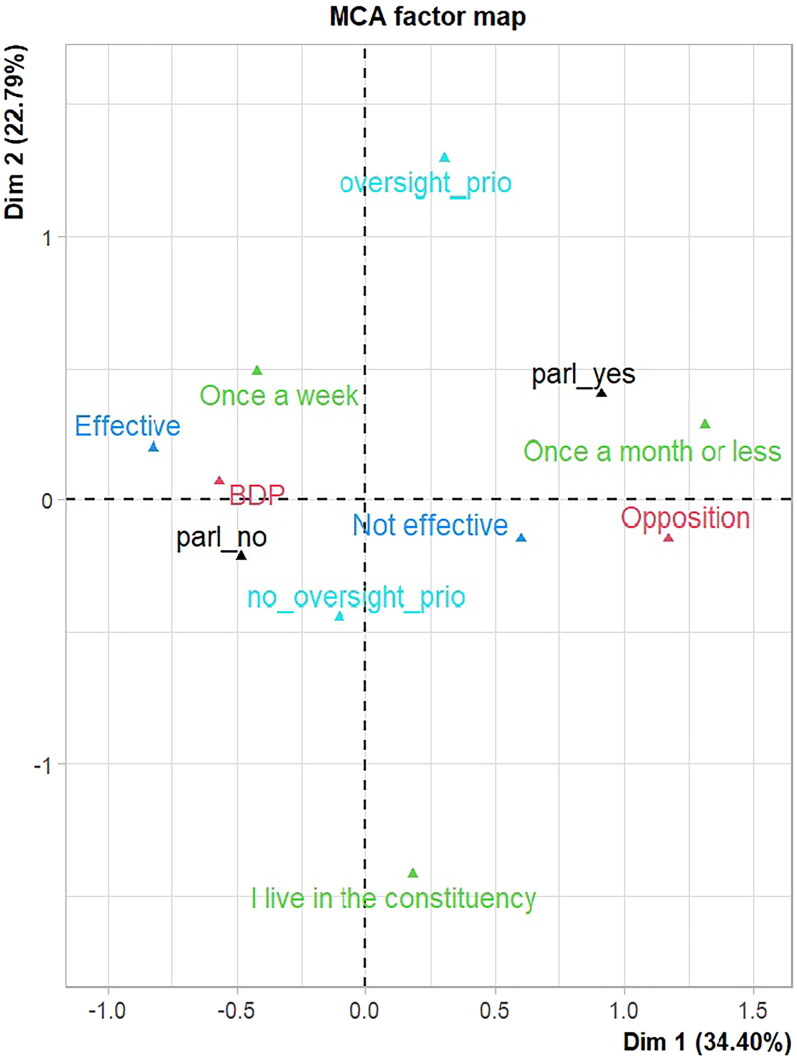
Factor plot from multiple correspondence analysis, showing how the categories are related to each other.

The following plots will show the associations between variable categories (Figure 3) and the profiles of individuals (Figure 5). MCA calculates the distances between the variable categories: the closer they are together, the closer their association is. Unsimilar categories are further apart on the plot. From the MCA plot, shown in Figure 3, we learn that MPs who talk about parliamentary affairs tend to belong to the opposition rather than the BDP, which has already been shown in the network graph in Figure 1. Furthermore, they are more critical of parliamentary effectiveness, visit their home constituency less frequently, and are more interested in parliamentary oversight.

Figure 3 shows the factor plot from MCA. Dimension 1 explains 22.79 per cent of the variance, and Dimension 2 explains 34.40 per cent of the variance. Together, the two dimensions explain about 57 per cent of the total variability in the dataset (this is called inertia in MCA).^95^ The interpretation is thus limited to the first two dimensions. Individuals who talk about parliamentary affairs are located in the upper right quadrant. They have positive values for both Dimension 1 and 2, whereas individuals who do not talk about parliamentary affairs are located in the lower left quadrant with negatives values on both axes. The individuals with positive coordinates for Dimension 1 share the following characteristics: high frequency for the factors *party*=Opposition, *talkparl*=parl_yes, *effectiveness*=Not effective, *goinghome*=Once a month or less and *oversight*=oversight_prio. Individuals with negative coordinates on this axis share characteristics like *party*=BDP, *talkparl*=parl_no, *goinghome*=Once a week, *oversight*=no_oversight_prio and *effectiveness*=Effective. Dimension 2 separates individuals with different values on the variable *oversight* and *goinghome*.

**Figure 4.**
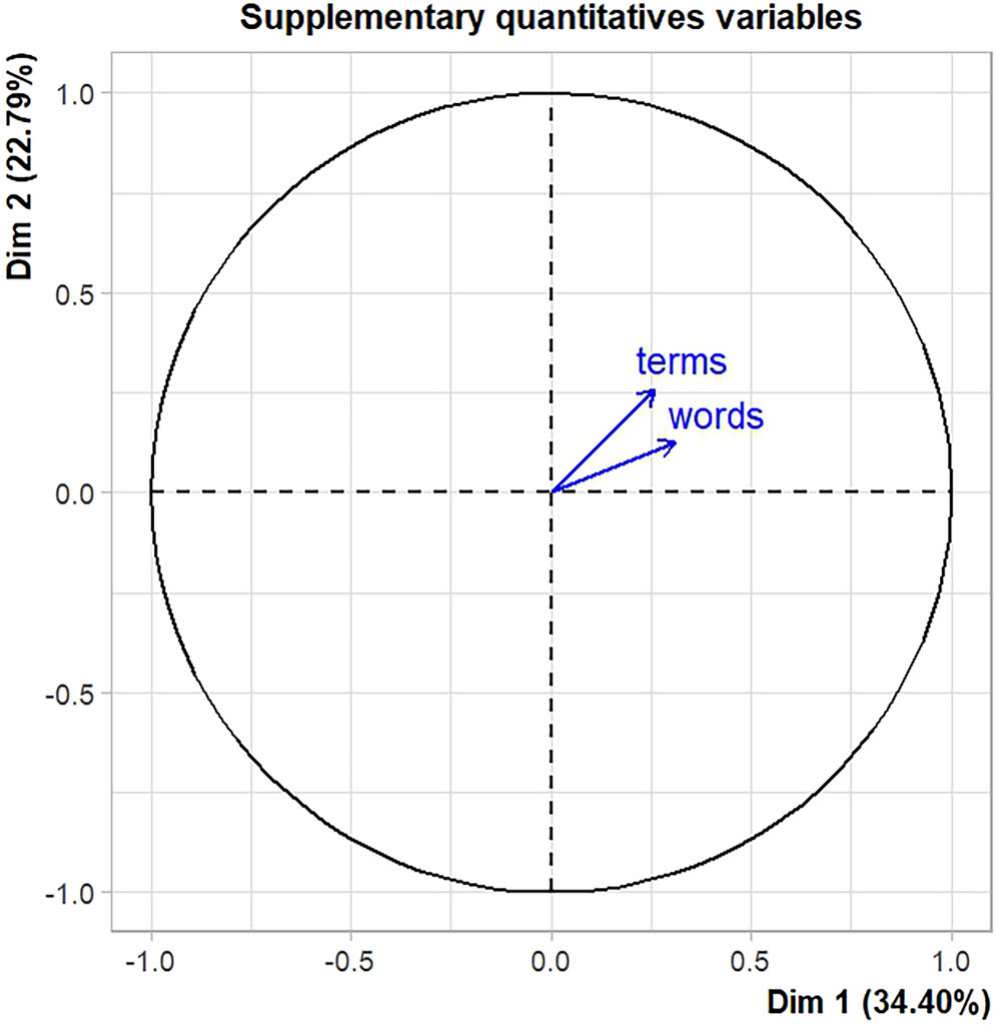
Analysis of supplementary quantitative variables: the location of the variables *words* and *terms* in the upper right quadrant shows that the group which talks about parliamentary affairs is also more active in parliament and has more experience.

Our analysis also reveals that the group which talks about parliamentary affairs is also more active in parliament and has more experience. The location of the two supplementary quantitative variables *words* and *terms* in the Euclidean space is shown in Figure 4. Both are found in the upper right quadrant, which is characterised by MPs who have a high priority in oversight, go home only one a month or less, and talk about parliamentary affairs, as shown in Figure 3.

**Figure 5.**
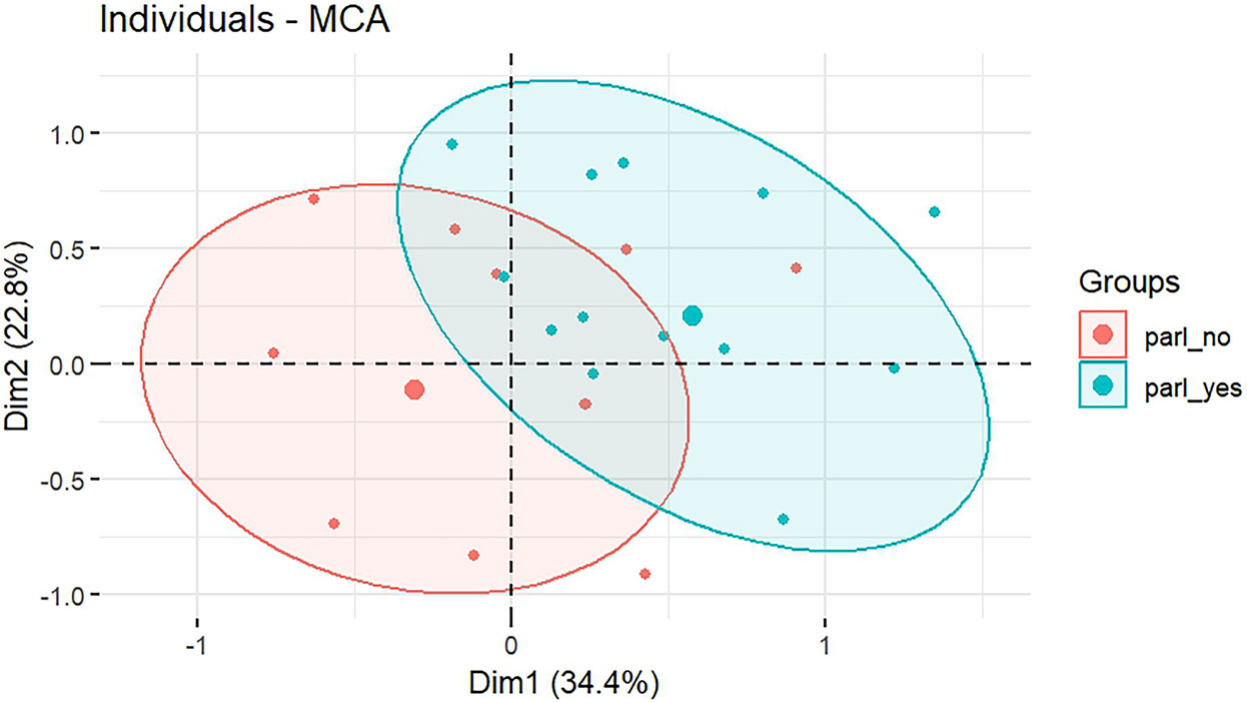
Plot of individuals based on multiple correspondence analysis, showing the MPs in parliament. Ellipses are drawn around MPs who talk about parliamentary affairs or do not talk about parliamentary affairs.

Figure 5 maps the individuals in the same Euclidean space. Again, the more similar individuals are, the closer they are in the plot. Almost all MPs who talk about parliamentary affairs are found in the upper right quadrant. They are coloured in green in the graph. Many, but not all, of the MPs who do *not* talk about parliamentary affairs are found in the lower left quadrant. They are coloured in red. Some of the dots on the graph are bigger than others; this means that more than one MP is characterised by the same combination of attributes and hence located in the same space. To visualise the difference between MPs talking/not talking about parliamentary affairs, an ellipse is drawn around the groups. There is some overlap towards the centre of the graph, otherwise the groups of MPs are relatively separated from each other.

We can conclude that our coalition for change tends to be composed by MPs who hold critical views on parliamentary effectiveness. They are more interested in parliamentary business than in constituency service and they also speak more often on the floor. While it is not surprising that they tend to belong to the opposition rather than the ruling party, the result on the number of terms is interesting and runs counter to our expectations. Barkan argued that reformers are often voted out because of their lack of engagement in constituency service. Although statistically not significant, we find the opposite: members in the coalition for change tend to be more slightly more experienced. This can have several reasons. Perhaps MPs who have served longer in parliament tend to become more critical on parliamentary business because they understand more about the procedures and shortcomings of parliamentary business. At least in our data this does not lead them to be voted out of office. These relationships can be further explored in other future studies.

To further confirm the relationships between the variables, we conducted an additional statistical test. Using Exponential Random Graph Models (ERGMs), which are specialised statistical models for social network data,^96^ we find that members of a coalition for change have a significantly higher probability of belonging to the opposition. They are also significantly less likely to visit their home constituency. While they are also more interested in oversight, the effect is not significant. Detailed results can be found in the Appendix.

## Conclusion

In this article, we have explored informal communication structures in the Parliament of Botswana. Starting from the idea that different MPs have different role orientations, we have theoretically and empirically advanced Barkan’s concept of a ‘coalition for change’. Conventional explanations of Botswana’s parliament depict it as a weak institution, ineffective in law making and in exercising oversight on the executive. Generally, these studies present anecdotal evidence about the need for reform, albeit with no success.

Our study, however, finds evidence for an emerging coalition for change. First of all, we see a number of MPs that are connected through their interest in the topic of parliamentary affairs. These MPs are to large degree connected, but there are smaller subnetworks. An established communication structure is a facilitator of collective action. Once formed, these connections can be used to pushed for specific issues or to initiate reforms. There is no guarantee, however, that this will be successful. We therefore prefer to emphasise the latent possibility of reform initiatives on the grounds of existing personal networks. Opposition MPs are more likely to be interested in parliamentary affairs. Some BDP MPs joined the opposition-dominated network, but there is also a separate clique of ruling party MPs. Frequent home visits are negatively related to interest in parliamentary affairs. This points to differences in role orientation: while some MPs are more assiduous in constituency service, others focus more on speaking on the floor.

The findings emerging from this article contribute to a better understanding of Botswana’s parliamentary system. They are, to a large extent, in line with Barkan’s theory. Role orientations are a fruitful avenue for further research on African parliaments. Some MPs are more inclined than others to their formal duties like law-making duties and executive oversight. The fact that they tend to have served more terms raises the question of whether constituency service is really the decisive factor for re-election. Our data cannot answer this question, but they point to the need for further research on the determinants and consequences of role orientation. It should be borne in mind, however, that our data collection took place before the most decisive election in Botswana’s history. While we cannot say that the 2024 election result would have been predicted from our data, the cohesion among the opposition MPs and their social interaction structure might point to the role of communication for opposition coordination. Increasing dissatisfaction and the need for change could have inspired discussions about reform. What is also positive is that there are links between MPs on both sides of the political divide, which shows a general willingness to talk to each other. The opposition is nevertheless instrumental in the push for reform.

These findings hold several implications for the functioning of parliament in Botswana and the way scholars theorise the role of parliament. Theoretically, the findings of this article suggest that scholars need to broaden their lens in analysing parliaments by going beyond structural explanations to consider the role orientations of MPs. Empirically, we show that political outcomes of Botswana’s parliament broadly reflect differing role orientations held by MPs, with few MPs committed to parliamentary affairs while the majority care about constituency service. Therefore, while analysts emphasise the need for reforms that strengthen and enhance the functioning of parliament to make it more effective in law making and oversight, these may be necessary but not sufficient conditions. Reforms that address role orientations of MPs and more generally the functions of parliament in a democratic society are equally important. While this article does not denigrate constituency service, if MPs increasingly see this as their primary responsibility then law making and executive oversight may be compromised. Crucially, with the mixed (mis)understanding of what constituency service means, demands and pressures for constituency service as a fundamental role of MPs may give an impetus to clientelism seen elsewhere in African parliaments. By implication, law making and executive oversight stand in jeopardy. Surely, the quality of Botswana’s parliamentary democracy rests on effective institutions and parliament lies at the core.

## Footnotes

1 M.S. Fish, ‘Stronger Legislatures, Stronger Democracies’, *Journal of Democracy*, 17, 1 (2006), pp. 5–20;

2 *Ibid.*; see also M.S. Fish and M. Kroenig, *The Handbook of National Legislatures: A Global Survey* (New York, Cambridge University Press, 2009).

3 J.D. Barkan, *Legislative Power in Emerging African Democracies* (Boulder, Lynne Rienner Publishers, 2009).

4 J.D. Barkan, ‘African Legislatures and the “Third Wave” of Democratization’, in J.D. Barkan (ed.), *Legislative Power in Emerging African Democracies* (Boulder, Lynne Rienner Publishers, 2009), pp. 1–32, https://doi.org/10.1515/9781626371118-002.

5 *Ibid*.

6 The edited volume *Legislative Power in Emerging African Democracies* was published in 2009. It contains Barkan’s guiding theoretical chapter, in which he developed his main arguments. The book’s chapters dealing with the individual countries all refer to this theoretical framework.

7 Y.L. Morse, ‘The Legislature as Political Control: Change and Continuity in Cameroon’s National Assembly (1973–2019)’, *The Journal of Modern African Studies*, 59, 4 (2021), pp. 485–506, https://doi.org/10.1017/S0022278X21000288; K.O. Opalo, *Legislative Development in Africa: Politics and Postcolonial Legacies*, 1st edition (New York, Cambridge University Press, 2019); M. Collord, ‘Wealth, Power and Institutional Change in Tanzania’s Parliament’, *African Affairs*, 121, 482 (2022), pp. 1–28, https://doi.org/10.1093/afraf/adac008; L. Demarest, ‘Elite Clientelism in Nigeria: The Role of Parties in Weakening Legislator–Voter Ties’, *Party Politics*, 28, 5 (2022), pp. 939–53, https://doi.org/10.1177/13540688211030219; A. Osei and D. Wigmore-Shepherd, ‘Personal Power in Africa: Legislative Networks and Executive Appointments in Ghana, Togo and Gabon’, *Government and Opposition*, 59, 1 (2024), pp. 272–96, https://doi.org/10.1017/gov.2022.42.

8 N. Cheeseman, ‘Introduction: Understanding African Politics: Bringing the State Back In’, in N. Cheeseman (ed.), *Institutions and Democracy in Africa: How the Rules of the Game Shape Political Developments* (Cambridge, Cambridge University Press, 2018), pp. 1–38.

9 M. van Vliet, ‘Weak Legislatures, Failing MPs, and the Collapse of Democracy in Mali’, *African Affairs*, 113, 450 (2014), pp. 45–66, https://doi.org/10.1093/afraf/adt071; E. Azevedo-Harman, ‘Parliaments and Citizens in Sub-Saharan Africa’, *The Journal of Legislative Studies*, 18, 3–4 (2012), pp. 419–40, https://doi.org/10.1080/13572334.2012.706054; S.I. Lindberg, ‘What Accountability Pressures Do MPs in Africa Face and How Do They Respond? Evidence from Ghana’, *The Journal of Modern African Studies*, 48, 1 (2010), pp. 117–42, https://doi.org/10.1017/S0022278X09990243.

10 Barkan, *Legislative Power in Emerging African Democracies*; R. Mattes and S. Mozaffar, ‘Legislatures and Democratic Development in Africa’, *African Studies Review*, 59, 3 (2016), pp. 201–15, https://doi.org/10.1017/asr.2016.83.

11 A. Osei, ‘Vicious Cycles: Candidate Selection, Vertical Accountability, and MPs’ Performance in Sierra Leone’, *Africa Today*, 68, 3 (2022), pp. 109–29, https://doi.org/10.2979/africatoday.68.3.06.

12 Mattes and Mozaffar, ‘Legislatures and Democratic Development in Africa’.

13 J.W. Paller, ‘Informal Institutions and Personal Rule in Urban Ghana’, *African Studies Review*, 57, 3 (2014), pp. 123–42, https://doi.org/10.1017/asr.2014.95; B. Driscoll, ‘Democratization, Party Systems, and the Endogenous Roots of Ghanaian Clientelism’, *Democratization*, 27, 1 (2020), pp. 119–36, https://doi.org/10.1080/13510347.2019.1666265.

14 M. Acheampong, ‘Legislators’ Pathway to Power in Ghana: Intra-Party Competition, Clientelism and Legislator–Constituents’ Relationship’, *The Journal of Legislative Studies*, 27, 2 (2021), pp. 300–16, https://doi.org/10.1080/13572334.2020.1814514.

15 N. Ichino and N.L. Nathan, ‘Primaries on Demand? Intra-Party Politics and Nominations in Ghana’, *British Journal of Political Science*, 42, 4 (2012), pp. 769–91, https://doi.org/10.1017/S0007123412000014; N. Ichino and N.L. Nathan, ‘Democratizing the Party: The Effects of Primary Election Reforms in Ghana’, *British Journal of Political Science*, 52, 3 (2022), pp. 1168–85, https://doi.org/10.1017/S0007123421000028.

16 Demarest, ‘Elite Clientelism in Nigeria’.

17 J.A. Harris and D.N. Posner, ‘(Under What Conditions) Do Politicians Reward Their Supporters? Evidence from Kenya’s Constituencies Development Fund’, *The American Political Science Review*, 113, 1 (2019), pp. 123–39, https://doi.org/10.1017/S0003055418000709.

18 *Ibid*.

19 *Ibid*., p. 137.

20 Demarest, ‘Elite Clientelism in Nigeria’.

21 Collord, ‘Wealth, Power and Institutional Change in Tanzania’s Parliament’.

22 K.O. Opalo, ‘Constrained Presidential Power in Africa? Legislative Independence and Executive Rule Making in Kenya, 1963–2013’, *British Journal of Political Science*, 50, 4 (2020), pp. 1341–58, https://doi.org/10.1017/S0007123418000492.

23 J. Katomero, R. Hoppe and A. Wesselink, ‘Tales of Accountability: A Q-Method Study of Discourses amongst Tanzanian Members of Parliament’, *The Journal of Modern African Studies*, 55, 3 (2017), pp. 423–53, https://doi.org/10.1017/S0022278X1700009X.

24 C. Adida, J. Gottlieb, E. Kramon and G. McClendon, ‘When Does Information Influence Voters? The Joint Importance of Salience and Coordination’, *Comparative Political Studies*, 53 (2020), pp. 851–91, https://doi.org/10.1177/0010414019879945.

25 J.C. Wahlke, W. Buchanan, H. Eulau and L.C. Ferguson, ‘American State Legislators’ Role Orientations Toward Pressure Groups’, *The Journal of Politics*, 22, 2 (1960), pp. 203–27, https://doi.org/10.2307/2127356.

26 T. Saalfeld and W.C. Müller, ‘Roles in Legislative Studies: A Theoretical Introduction’, *The Journal of Legislative Studies*, 3, 1 (1997), pp. 1–16, https://doi.org/10.1080/13572339708420496.

27 K. Strøm, ‘Rules, Reasons and Routines: Legislative Roles in Parliamentary Democracies’, *The Journal of Legislative Studies*, 3, 1 (1997), pp. 155–74, https://doi.org/10.1080/13572339708420504.

28 S. Gherghina, ‘MPs’ Role Orientations in Newly Emerged Democratic Legislatures’, *Journal of Legislative Studies*, 17, 1 (2011), pp. 45–64, https://doi.org/10.1080/13572334.2011.545179.

29 Barkan, *Legislative Power in Emerging African Democracies*.

30 Barkan, ‘African Legislatures and the “Third Wave” of Democratization’.

31 J.D. Barkan, ‘Conclusion’, in J.D. Barkan (ed.), *Legislative Power in Emerging African Democracies* (Boulder, Lynne Rienner Publishers, 2009), pp. 231–50, https://doi.org/10.1515/9781626371118-009.

32 *Ibid*., p. 233.

33 *Ibid*.

34 *Ibid*.

35 *Ibid*., p. 232.

36 Barkan, ‘Conclusion’, p. 231.

37 L. Adamolekun and M. Laleye, ‘Benin: Legislative Development in Africa’s First Democratizer’, Chapter 4 in J.D. Barkan (ed.), *Legislative Power in Emerging African Democracies* (Boulder, Lynne Rienner Publishers, 2009), pp. 109–46, https://doi.org/10.1515/9781626371118-005.

38 *Ibid*.

39 P.M. Lewis, ‘Rules and Rents in Nigeria’s National Assembly’, Chapter 6 in J.D. Barkan (ed.), *Legislative Power in Emerging African Democracies* (Boulder, Lynne Rienner Publishers, 2009), pp. 177–204, https://doi.org/10.1515/9781626371118-007.

40 *Ibid*.

41 S.I. Lindberg and Y. Zhou, ‘Co-Optation Despite Democratization in Ghana’, Chapter 5 in J.D. Barkan (ed.), *Legislative Power in Emerging African Democracies* (Boulder, Lynne Rienner Publishers, 2009), pp. 147–76, https://doi.org/10.1515/9781626371118-006.

42 J.D. Barkan and Fred Matiangi, ‘Kenya’s Tortuous Path to Successful Legislative Development’, Chapter 2 in J.D. Barkan (ed.), *Legislative Power in Emerging African Democracies* (Boulder, Lynne Rienner Publishers, 2009), pp. 33–72, https://doi.org/10.1515/9781626371118-003.

43 Collord, ‘Wealth, Power and Institutional Change in Tanzania’s Parliament’.

44 Opalo, *Legislative Development in Africa*.

45 A. Poteete, ‘Renegotiation of Executive Powers and Executive-Legislative Relations in Botswana’, APSA 2010 Annual Meeting Paper, *SSRN Electronic Journal*, 1 January 2013, https://doi.org/10.2139/ssrn.3537279.

46 H. Best and L. Vogel, ‘The Sociology of Legislatures and Legislators’, in S. Martin, T. Saalfeld and K.W. Strøm (eds), *The Oxford Handbook of Legislative Studies* (Oxford, Oxford University Press, 2014), pp. 57–81; see also A. Osei, ‘Post-Conflict Democratisation in Sierra Leone: The Role of the Parliament’, *The Journal of Legislative Studies*, 27, 1 (2021), pp. 112–35, https://doi.org/10.1080/13572334.2020.1809806.

47 B. Tsie, ‘The Political Context of Botswana’s Development Performance’, *Journal of Southern African Studies*, 22, 4 (1996), pp. 599–616, https://doi.org/10.1080/03057079608708514; D. Magang, *Delusions of Grandeur: Paradoxies and Ambivalences in Botswana’s Macroeconomic Firmament* (Gaborone, Print Media Consult, 2015); D. Sebudubudu and B.Z. Osei-Hwedie, ‘Pitfalls of Parliamentary Democracy in Botswana’, *Africa Spectrum*, 41, 1 (2006), pp. 35–53; J.D. Holm, ‘Curbing Corruption through Democratic Accountability: Lessons From Botswana’, in K.R. Hope and B.C. Chikulo (eds), *Corruption and Development in Africa: Lessons from Country Case Studies* (London, Palgrave Macmillan UK, 2000), pp. 288–304, https://doi.org/10.1057/9780333982440_16; A.I. Samatar, *An African Miracle: State and Class Leadership and Colonial Legacy in Botswana Development* (Portsmouth, Heinemann Educational Books, 1999); M.G. Molomo, ‘Democracy under Siege: The Presidency and Executive Powers in Botswana’, *Pula: Botswana Journal of African Studies*, 14, 1 (2000), pp. 95–108, https://ubrisa.ub.bw/handle/10311/897; B.Z. Osei-Hwedie, ‘Introspection, Retrospection, Prospects and Lessons’, in D. Sebudubudu, B.Z. Osei-Hwedie and B. Tsie (eds), *Botswana’s Parliamentary Democracy Revisited* (Pretoria, Unisa Press, 2017), pp. 230–7; K. Good, *Diamonds, Dispossession & Democracy in Botswana* (Woodbridge, Boydell & Brewer, 2008).

48 Z. Maundeni and K. Lotshwao, ‘Internal Organisation of Political Parties in Botswana’, *Global Journal of Human-Social Science*, 12, C9 (2012), pp. 55–63; W. Maathai, *The Challenge for Africa* (London, Arrow Books, 2010); D.L. Keorapetse, ‘“Monopoly Politikos”: An Account of a De Facto One-Party State and Lack of Regime Change in Botswana’, in M.N. Amutabi and S.W. Nasong’o (eds), *Regime Change and Succession Politics in Africa: Five Decades of Misrule* (New York, Routledge, 2012), pp. 217–37.

49 Molomo, ‘Democracy under Siege’; K. Good and I. Taylor, ‘Unpacking the “Model”: Presidential Succession in Botswana’, in R. Southall and H. Melber (eds), *Legacies of Power: Leadership Change and Former Presidents in African Politics* (Cape Town, HSRC Press, 2006), pp. 51–72; M.Z. Botlhomilwe and D. Sebudubudu, ‘Elections in Botswana: A Ritual Enterprise?’, *The Open Area Studies Journal*, 4, 1 (2011), pp. 96–103, https://doi.org/10.2174/1874914301104010096.

50 P. Molutsi, ‘Botswana’s Democracy in a Southern African Regional Perspective: Progress or Decline?’, in Z. Maundeni (ed.), *40 Years of Democracy in Botswana: 1965–2005* (Gaborone, Mmegi Publishing House, 2005), pp. 10–28.

51 D.D.N. Nsereko, *Constitutional Law in Botswana* (Gaborone, Pula Press, 2002).

52 B. Tsie, ‘Botswana’s Developmental Democracy in a Global Context’, in D. Sebudubudu, B.Z. Osei-Hwedie and B. Tsie (eds), *Botswana’s Parliamentary Democracy Revisited* (Pretoria, Unisa Press, 2017), pp. 181–201.

53 Nsereko, *Constitutional Law in Botswana*, pp. 139–49.

54 E.K. Botlhale, ‘Accountability and Democracy’, in K. Alexander and G. Kaboyakgosi (eds), *A Fine Balance. Assessing the Quality of Governance in Botswana* (Pretoria, IDASA, 2012), pp. 53–64; Nsereko, *Constitutional Law in Botswana*.

55 G. Barei, ‘Parliament’, in Z. Maundeni (ed.), *Transparency, Accountability & Corruption in Botswana* (Gaborone, IDASA, 2008), pp. 11–25.

56 Holm, ‘Curbing Corruption through Democratic Accountability’; Sebudubudu and Osei-Hwedie, ‘Pitfalls of Parliamentary Democracy in Botswana’; Barei, ‘Parliament’; K. Bodilenyane, ‘Botswana’s Executive Presidency: Implications for Democracy’, *Journal of Public Administration and Governance*, 2, 4 (2012), pp. 188–201, https://doi.org/10.5296/jpag.v2i4.2749; E.K. Botlhale and K. Lotshwao, ‘The Uneasy Relationship Between Parliament and the Executive in Botswana’, *Botswana Notes and Records*, 45 (2015), pp. 39–51, available at https://journals.ub.bw/index.php/bnr/article/view/395, retrieved 22 February 2025.

57 Barkan, ‘Conclusion’.

58 Barkan, *Legislative Power in Emerging African Democracies*.

59 See, among others, B.Z. Osei-Hwedie, ‘The Political Opposition in Botswana: The Politics of Factionalism and Fragmentation’, *Transformation: Critical Perspectives on Southern Africa*, 45 (2001), pp. 57–77; B. Seabo and B. Masilo, ‘Social Cleavages and Party Alignment in Botswana: Dominant Party System Debate Revisited’, *Botswana Notes and Records*, 50 (2018), pp. 59–71; Keorapetse, ‘Monopoly Politikos’; Z. Kebonang and G. Kaboyakgosi, ‘The Political Party System: Explaining the Predominance of the Botswana Democratic Party’, in D. Sebudubudu, B.Z. Osei-Hwedie and B. Tsie (eds), *Botswana’s Parliamentary Democracy Revisited* (Pretoria, Unisa Press, 2017), pp. 66–77.

60 Molutsi, ‘Botswana’s Democracy in a Southern African Regional Perspective’, p. 21.

61 K. Good, ‘The State and Extreme Poverty in Botswana: The San and Destitutes’, *The Journal of Modern African Studies*, 37, 2 (1999), pp. 185–205, https://doi.org/10.1017/S0022278X99003043.

62 K. Good, ‘Accountable to Themselves: Predominance in Southern Africa’, *The Journal of Modern African Studies*, 35, 4 (1997), pp. 547–73.

63 Sebudubudu and Osei-Hwedie, ‘Pitfalls of Parliamentary Democracy in Botswana’.

64 R. Molomo, *Democratic Deficit in the Parliament of Botswana* (Cape Town, Centre for Advanced Studies of African Society, 2012).

65 Sebudubudu and Osei-Hwedie, ‘Pitfalls of Parliamentary Democracy in Botswana’.

66 Molomo, *Democratic Deficit in the Parliament of Botswana*.

67 Botlhale and Lotshwao, ‘The Uneasy Relationship Between Parliament and the Executive in Botswana’.

68 Mattes and Mozaffar, ‘Legislatures and Democratic Development in Africa’.

69 Nsereko, *Constitutional Law in Botswana*.

70 Sebudubudu and Osei-Hwedie, ‘Pitfalls of Parliamentary Democracy in Botswana’, p. 39.

71 Barei, ‘Parliament’; M.N. Nasha, *Madam Speaker, Sir!: Breaking the Glass Ceiling: One Woman’s Struggles* (Gaborone, Diamond Educational Publishers, 2014); Bodilenyane, ‘Botswana’s Executive Presidency’.

72 C.M. Fombad, ‘The Separation of Powers and Constitutionalism in Africa: The Case of Botswana’, *Boston College Third World Law Journal*, 25, 2 (2005), pp. 301–25.

73 M. Mogalakwe and F. Nyamnjoh, ‘Botswana at 50: Democratic Deficit, Elite Corruption and Poverty in the Midst of Plenty’, *Journal of Contemporary African Studies*, 35, 1 (2017), pp. 1–14, 4.

74 Botlhale and Lotshwao, ‘The Uneasy Relationship Between Parliament and the Executive in Botswana’.

75 *Ibid*.

76 Nasha, *Madam Speaker, Sir!*

77 Sebudubudu and Osei-Hwedie, ‘Pitfalls of Parliamentary Democracy in Botswana’; Molomo, *Democratic Deficit in the Parliament of Botswana*.

78 D. Sebudubudu and M.Z. Botlhomilwe, ‘Interrogating the Dominant Party System in Botswana’, in N. de Jager and P. du Toit (eds), *Friend or Foe? Dominant Party Systems in Southern Africa* (Claremont, UCT Press, 2013), https://doi.org/10.58331/UCTPRESS.9.

79 Barkan, ‘African Legislatures and the “Third Wave” of Democratization’, p. 18.

80 M. Galeragwe, ‘Parliament Passes National Assembly Salaries and Allowances Bill’, *Daily News*, Gaborone, 17 December 2023, available at https://dailynews.gov.bw/news-detail/77086, retrieved 22 February 2025.

81 Notably, Dumelang Saleshando, MP for Maun West, proposed an amendment to the bill, specifically that the president should not be entitled to a constituency allowance as he does not have a constituency and also because his trips are fully catered for by government in terms of food, transport, accommodation and fuel: see Galeragwe, ‘Parliament Passes National Assembly Salaries and Allowances Bill’.

82 L. Mpuang, ‘New MPs Want an Independent Parliament’, *The Botswana Gazette*, Gaborone, 13 November 2019, available at https://www.thegazette.news/news/new-mps-want-an-independent-parliament/retrieved 3 March 2025.

83 J.S. Levy, ‘Case Studies: Types, Designs, and Logics of Inference’, *Conflict Management and Peace Science*, 25, 1 (2008), pp. 1–18. http://doi.org/10.1080/07388940701860318.

84 We trained eight research assistants who then conducted face-to-face interviews in 2022. A research permit had to be obtained from the Parliament of Botswana, and each MP had to give his or her individual informed consent. Data were collected with tablet computers and personal data were stored according to a data safety protocol.

85 For some core characteristics of the sample, please see the Appendix.

86 U. Brandes and D. Wagner, ‘Visone – Analysis and Visualization of Social Networks’, in M. Jünger and P. Mutzel (eds), *Graph Drawing Software* (Berlin, Springer-Verlag, 2004), pp. 321–40, available at https://link.springer.com/book/10.1007/978-3-642-18638-7, retrieved 22 February 2025.

87 M.S. Handcock, D.R. Hunter, C.T. Butts, S.M. Goodreau and M. Morris, ‘Statnet: Software Tools for the Representation, Visualization, Analysis and Simulation of Network Data’, *Journal of Statistical Software*, 24, 1 (2008), https://doi.org/10.18637/jss.v024.i01.

88 Parliament of Botswana, ‘What is a Hansard?’, with ‘Search all Hansard’ facility, available at https://www.parliament.gov.bw/index.php?option=com_documents&view=files&catid=87&Itemid=438. In our search, we used 2019, because this year falls into the electoral period that we cover in our data collection. It was the last year in which parliamentary proceedings were face to face before Covid-19 forced the parliament into online sessions in 2020.

89 J.P. Benzecri, *Correspondence Analysis Handbook*, 1st edition (Boca Raton, CRC Press, 1992).

90 F. Husson, J. Josse, S. Le and J. Mazet, ‘FactoMineR: Multivariate Exploratory Data Analysis and Data Mining’, publication date 29 February 2024, https://cran.r-project.org/web/packages/FactoMineR/index.html.

91 P. Vaissie, A. Monge and F. Husson, ‘Factoshiny: Perform Factorial Analysis from “FactoMineR” with a Shiny Application’, publication date 20 November 2023, https://cran.r-project.org/web/packages/Factoshiny/index.html.

92 M.J. Greenacre, ‘Interpreting Multiple Correspondence Analysis’, *Applied Stochastic Models and Data Analysis*, 7, 2 (1991), pp. 195–210, https://doi.org/10.1002/asm.3150070208, p. 197; Benzecri, *Correspondence Analysis Handbook*, p. 44.

93 Greenacre, ‘Interpreting Multiple Correspondence Analysis’, p. 197.

94 M.J. Greenacre, ‘Biplots in Correspondence Analysis’, *Journal of Applied Statistics*, 20, 2 (1993), pp. 251–69, https://doi.org/10.1080/02664769300000021.

95 The value is greater than the reference value of 49.03 per cent. This is calculated from 10,000 simulated data tables of equivalent size and gives the 0.95-quantile of the inertia percentages distribution. The interpretation is that significant variability is explained in our model.

96 D.R. Hunter, M.S. Handcock, C.T. Butts, S.M. Goodreau and M. Morris, ‘Ergm: A Package to Fit, Simulate and Diagnose Exponential-Family Models for Networks’, *Journal of Statistical Software*, 24, 3 (2008), nihpa54860, https://www.jstatsoft.org/article/view/v024i03.

## Appendix

### 1 Variables

We use the following variables:

- *Talkparl* is a variable that is constructed from the discussion network. It is coded as ‘yes’ for MPs who are part of a dyad that talks about parliamentary affairs, and ‘no’ for all others. Eighteen MPs are involved in those discussions, either initiating them or being mentioned as a discussion partner.
- *party* is a binary variable with two categories, BDP and opposition. We do not distinguish between individual parties, because some opposition parties are very small. Moreover, in a dominant party system like that of Botswana the main cleavage in the party system is between the ruling party and the opposition.
- *terms* is a continuous variable which gives the number of terms that an MP has served in parliament. It runs from 1 (newcomers) to 3.
- *effectiveness* measures MPs’ perception of parliamentary effectiveness with four categories: ‘very effective’, ‘somewhat effective’, ‘not very effective’ and ‘not at all effective’. This is based on a survey question asking MPs to evaluate the effectiveness of parliament in executive oversight. For this article, the positive and negative categories were collapsed into one respectively, giving two levels: ‘effective’ and ‘not effective’.
- *oversight* is a variable that measures the preferences of MPs with regard to their role orientations. MPs were asked to rank a number of tasks that MPs typically fulfil by their importance. The items are: bringing development to the constituency; executive oversight; lawmaking; being close to the people; and giving feedback to constituents. The variable *oversight* has two levels: it is coded as oversight_prio if the MP has ranked executive oversight as the most important or second most important item, and as no_oversight_prio if not.
- *goinghome* is a proxy for constituency service. It is based on a survey question that measures the frequency with which MPs visit their constituency.
- *words* is a count of the number of words an MP has spoken on the record during the parliamentary year 2019. It is calculated from Hansard records that are available on the website of the Parliament of Botswana at: https://www.parliament.gov.bw/index.php?option=com_documents&view=files&catid=87&Itemid=438. We use 2019, because this year falls into the electoral period that we cover in our data collection. It was the last year in which parliamentary proceedings were face to face before Covid-19 forced the parliament into online sessions in 2020.

### 2 Further statistical tests

We undertook some further empirical tests. In these, we seek in particular to predict how the ties shown in Figure 2 are formed. The variables are the same as in the MCA (multiple correspondence analysis). ERGMs (Exponential Random Graph Models) take the network itself as the dependent variable and model the probability that a tie is formed. In other words, the model predicts membership in a coalition for change that is defined as network of people discussing parliamentary affairs. The results are displayed in Table 1.

**Table 1.** Empirical tests modelling the probability that ties between MPs are formed

	Model 1	Model 2	Model 3
Edges	2.42 (1.96)	1.18 (2.62)	–2.11 (2.69)
Frequency of home visits	–0.68 (0.22)**	–0.61 (0.23)**	–0.52 (0.22)*
Parliamentary effectiveness	–0.28 (0.18)	–0.25 (0.19)	0.03 (0.21)
Oversight	0.12 (0.46)	0.05 (0.47)	–0.02 (0.49)
Number of words		–0.00 (0.00)	0.00 (0.00)
Number of terms		0.24 (0.28)	0.10 (0.26)
Opposition			1.39 (0.48)**
AIC	189.31	192.59	185.36
BIC	212.85	227.88	226.54

*** p < 0.001; **p < 0.01; *p < 0.05; ^+^p < 0.1

The term ‘Edges’ measures the baseline probability of a tie being formed. AIC (Akaike Information Criterion) and BIC (Bayesian Information Criterion) are measures of model fit, and lower values for these indicate a better fit to the data. Model 1 includes the variables *goinghome*, *effectiveness* and *oversight*. Increasing frequencies of home visits significantly reduce the likelihood of participating in exchanges on parliamentary affairs. This result holds true in all three models. The other variables do not reach statistical significance. Model 2 adds *words* and *terms*, which are not statistically significant. Model 3 adds *Opposition*, which gives a positive and large significant effect.

While the MCA analysis has shown how the variables relate to each other, the statistical analysis finds significant effects for members of the opposition and the frequency of visiting the constituency.

